# Few-Mode Fiber with Low Spontaneous Raman Scattering for Quantum Key Distribution and Classical Optical Communication Coexistence Systems

**DOI:** 10.3390/s24237645

**Published:** 2024-11-29

**Authors:** Qi Zhao, Jianjun Tang, Weiwen Kong, Zhenyu Zhao, Jingjing Zheng, Yang Liu

**Affiliations:** 1 Institute of Basic Operations Technology, China Telecom Research Institute, Beijing 102209, China; tangjj6@chinatelecom.cn (J.T.); kongww1@chinatelecom.cn (W.K.); liuyang19@chinatelecom.cn (Y.L.); 2 China Academy of Information and Communications Technology, Beijing 102209, China; zhaozhenyu@caict.ac.cn; 3 Key Laboratory of All Optical Network and Advanced Telecommunication Network of Ministry of Education, Beijing Jiaotong University, Beijing 100044, China; jjzheng@bjtu.edu.cn

**Keywords:** quantum key distribution, few-mode fiber, spontaneous Raman scattering

## Abstract

In this paper, the theoretical model of spontaneous Raman scattering (SpRS) in few-mode fiber (FMF) is discussed. The influence of SpRS on quantum key distribution (QKD) in FMF is evaluated by combining wavelength division multiplexing (WDM) and space division multiplexing (SDM) techniques. On this basis, an improved ring-assisted FMF is designed and characterized; the transmission distance can be increased by up to 54.5% when choosing different multi-channels. The effects of forward and backward SpRS on QKD are also discussed.

## 1. Introduction

As a highly secure and reliable encryption method, QKD can generate secure keys between remote communication entities based on the inherent unpredictability of quantum physics [1]. It provides a theoretical basis for secure symmetric key encryption systems and has the potential to fundamentally alter the methods of protecting information exchange in the future. In the past few years, significant progress has been made in QKD research in terms of protocols and networks [2,3,4], including improvements to QKD protocols [5,6], noise analysis in quantum channels [7] and exploration of quantum relay networks [8,9]. The transmission distance and security key rate (SKR) of QKD have been greatly improved. In ultra-low-loss fiber, the transmission distance of QKD can reach up to 404 km [8], and the longest transmission distance of twin-field QKD is extended to 1002 km [10]. The highest SKR of high-speed QKD systems can reach 110 Mbps [11]. These advances have paved the way for the implementation of QKD networks. However, the high cost of deploying dedicated fibers is a major obstacle to the widespread use of QKD [12,13]. A promising solution to reduce deployment costs is to coexist QKD with classic optical communication. The coexistence system was first proposed in single-mode fiber (SMF) and has undergone a series of theoretical and experimental studies in WDM [14,15]. Nevertheless, the SpRS generated by classical signals is the biggest challenge of the scheme [16,17]. The wavelength range of Raman scattering exceeds 200 nm, making it susceptible to affecting quantum channels. It hinders the practical application of coexistence solutions in the most advanced technologies.

At the same time, in order to further improve the capacity, research on SDM has received increasing attention. Several types of optical fibers can achieve spatial multiplexing, such as multi-core fiber (MCF) [18], FMF, and few-mode multi-core fiber [19]. Among them, the modes supported by FMF can be used as parallel channels for independent signals. Compared with MCF, FMF has a simple manufacturing process and large effective mode field area, which is beneficial for reducing nonlinear noise and suitable for transmitting sensitive quantum signals [20,21]. Unfortunately, despite numerous experimental studies on FMF [22,23], there is limited theoretical research evaluating the impact of SpRS on QKD in FMF. Therefore, this paper explores the issue based on previous research. According to the conclusion, an improved ring-assisted FMF is designed, which significantly increases the signal transmission distance.

## 2. The SpRS When QKD Coexists with Classical Signals in FMF

In the coexistence system based on FMF, each mode only transmits classical or quantum signals, and the classical signals in each mode are at different wavelengths. This section will discuss the properties of the SpRS between modes in FMF and analyze its impact on the SKR of QKD systems.

### 2.1. Derivation of the SpRS in FMF

The proposed SpRS is based on the inter mode crosstalk effect. Firstly, the process of mode crosstalk is derived as shown in Equations (l) and (2), where *P_i_* and *P_j_* are the transmitted powers in each channel along the *z*-axis, *α* is the attenuation coefficient in the channels, and *h_ij_* is the power coupling coefficient [24].

(1) $$\frac{dP_{j}(z)}{dz}=-\alpha z+h_{ij}(P_{j}-P_{i})$$

(2) $$\frac{dP_{i}(z)}{dz}=-\alpha z+h_{ij}(P_{i}-P_{j})$$

Equations (3) and (4) can be obtained by solving Equations (1) and (2) for the conditions that *P_j_*(0) = *p_c_* and *P_i_*(0) = *p_q_*, which represent the initial power of the classical and quantum channel, respectively.

(3) $$P_{j}(z)=\frac{1}{2}\left[\left(p_{c}+p_{q})\cdot \exp(-\alpha z)-(p_{c}-p_{q})\cdot \exp(-(\alpha +h_{ij})z\right)\right]$$

(4) $$P_{i}(z)=\frac{1}{2}\left[\left(p_{c}+p_{q})\cdot \exp(-\alpha z)+(p_{c}-p_{q})\cdot \exp(-(\alpha +h_{ij})z\right)\right]$$

Then, the derivation of SpRS is carried out, that is, the classical signal of the *n*th wavelength channel in *j*th mode generates SpRS in the *m*th quantum channel of *i*th mode. The SpRS is mainly composed of two parts. One is that the classical signals generate crosstalk in the channel of the quantum signal, which produces SpRS. The other part is that the classical signal generates SpRS in its own channel, and then the SpRS crosstalk in the channel of the quantum signal.

In the first part, assuming the transmission distance is *dz*, the power of forward SpRS is as follows [12]:

(5) $$dPL_{ICXT-FSRS}(z)=\eta_{mn}P_{ICXT}(z)dz$$

where *η_mn_* is the Raman scattering factor between the *m*th and *n*th wavelength channels, and *P_ICXT_*(*z*) is the crosstalk power at *dz*, which can be obtained from Equation (4).

Due to channel loss, when the power of SpRS is transmitted to the output end of the fiber, the power can be obtained as shown in Equation (6). The equation can be integrated to obtain the power of the first part of the forward SpRS, as shown in Equation (7).

(6) $$dP_{ICXT-FSRS}(z)=dPL_{ICXT-FSRS}(z)\exp[-\alpha_{q}(L-z)]$$

(7) $$\begin{array}{cc} P_{ICXT-FSRS} & =\int_{0}^{L}dP_{ICXT-FSRS}(z)dz\\ & =\frac{1}{2}\eta_{mn}\cdot e^{-\alpha_{i}L}\cdot \left\{\frac{p_{c}+p_{q}}{\alpha_{i}-\alpha_{j}}[e^{(\alpha_{i}-\alpha_{j})L}-1]\right.\left.-\frac{p_{c}-p_{q}}{\alpha_{i}-\alpha_{j}-2h_{ij}}[e^{(\alpha_{i}-\alpha_{j}-2h_{ij})L}-1]\right\} \end{array}$$

A similar method is used to derive the second part of forward SpRS. The power of Raman scattering generated at the *dz* distance in *j*th mode is

(8) $$dPL_{FSRS-ICXT}(z)=\eta_{mn}P_{CS}(z)dz$$

*P_cs_*(*z*) is the power of the signal at *dz*, which can be obtained from Equation (3). The power crosstalks into the *i*th mode and transmits to the output port of the FMF, as shown in Equation (9).

(9) $$dP_{FSRS-ICXT}(z)=dPL_{FSRS-ICXT}(z)\exp[-\alpha_{q}(L-z)]$$

The power equation can be derived as [25,26]:

(10) $$\begin{array}{cc} P_{FSRS-ICXT} & =\int_{0}^{L}dP_{FSRS-ICXT}(z)dz\\ & =\frac{1}{2}\eta_{mn}\cdot e^{-\alpha_{i}L}\cdot \left\{\frac{p_{c}+p_{q}}{\alpha_{i}-\alpha_{j}}[e^{(\alpha_{i}-\alpha_{j})L}-1]\right.\left.-\frac{p_{c}-p_{q}}{\alpha_{i}-\alpha_{j}-2h_{ij}}[e^{(\alpha_{i}-\alpha_{j}-2h_{ij})L}-1]\right\} \end{array}$$

From the above derivation, it can be concluded that the forward SpRS is

(11) $$\begin{array}{cc} P_{FSRS} & =P_{ICXT-FSRS}+P_{FSRS-ICXT}\\ & =\eta_{mn}\cdot e^{-\alpha_{i}L}\cdot \left\{\frac{p_{c}+p_{q}}{\alpha_{i}-\alpha_{j}}[e^{(\alpha_{i}-\alpha_{j})L}-1]\right.\left.-\frac{p_{c}-p_{q}}{\alpha_{i}-\alpha_{j}-2h_{ij}}[e^{(\alpha_{i}-\alpha_{j}-2h_{ij})L}-1]\right\} \end{array}$$

For the case of multiple classical channels, the forward SpRS of the *m*th channel in *i*th mode can be calculated as

(12) $$P_{F\_SpRS}=\sum_{j=1}^{J}\sum_{n=1}^{N}P_{FSRS}^{j,n}$$

Similarly, the backward SpRS can be obtained as

(13) $$\begin{array}{cc} P_{BSRS} & =P_{ICXT-BSRS}+P_{BSRS-ICXT}\\ & =\eta_{mn}\cdot \left\{\frac{p_{c}-p_{q}}{\alpha_{i}+\alpha_{j}+2h_{ij}}[1-e^{-(\alpha_{i}+\alpha_{j}+2h_{ij})L}]\right.\left.+\frac{p_{c}+p_{q}}{\alpha_{i}+\alpha_{j}}[1-e^{-(\alpha_{i}+\alpha_{j})L}]\right\} \end{array}$$

For the case of multiple classical channels, the backward SpRS of the *m*th channel in *i*th mode can be calculated as

(14) $$P_{B\_SpRS}=\sum_{j=1}^{J}\sum_{n=1}^{N}P_{BSRS}^{j,n}$$

### 2.2. Impact of the SpRS on QKD

The protocol used in the simulation is the BB84 protocol with decoy-state method. The SKR is lower-bounded by [27]

(15) $$R=q\left\{-Q_{\mu }f_{e}H_{2}(E_{\mu })+Q_{1}[1-H_{2}(e_{1})]\right\}$$

where *H*_2_ is the binary Shannon entropy, *q* is set to 1/2 for the BB84 protocol, and *f_e_* accounts for the efficiency of error correction, which is set to 1.15. *μ* is the average number of photons in one pulse. *Q_μ_* and *E_μ_* are the overall gain and the quantum bit error rate. *Q*_1_ and *e*_1_ are the gain and the error rate of a single-photon state. *Q_μ_* and *E_μ_* can be obtained by *Y*_0_, which is the noise count including the dark count of the single photon detector and the SpRS noise. Considering the widely used dual-detector system, *Y*_0_ can be expressed as Ref. [12],

(16) $$Y_{0}=2p_{dark}+p_{SRS}$$

where *p_dark_* is the dark count rate of each single photon detector. *p_SRS_* is the noise photon caused by the SpRS, which can be calculated by [15].

(17) $$p_{SRS}=\left\{\begin{array}{c} \frac{P_{F\_SpRS}\times \Delta f\times \Delta t}{h\times f},\;forward\\ \frac{P_{B\_SpRS}\times \Delta f\times \Delta t}{h\times f},\;backward \end{array}\right.$$

where *h* is the Planck’s constant, and *f* is the frequency of the quantum channel. Δ*f* is the receiving bandwidth of the quantum channel, and Δ*t* is the detector effective gating width.

## 3. Performance of QKD in FMFs

**Figure 1:**
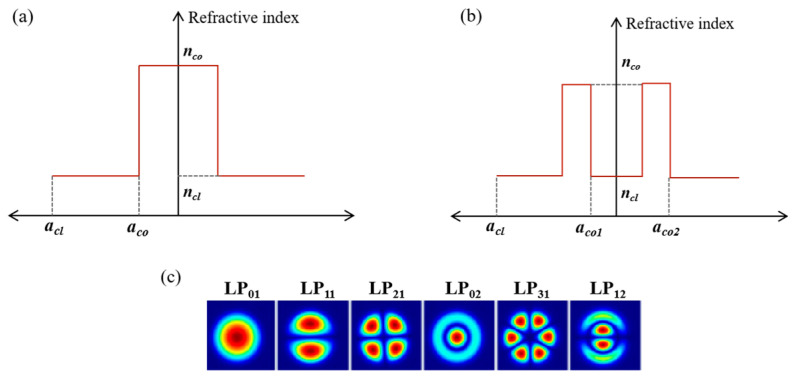
The radius and refractive index distribution of (**a**) step-index fiber and (**b**) ring-core fiber (RCF). (**c**) The modes that can be supported in fibers.

**Figure 2:**
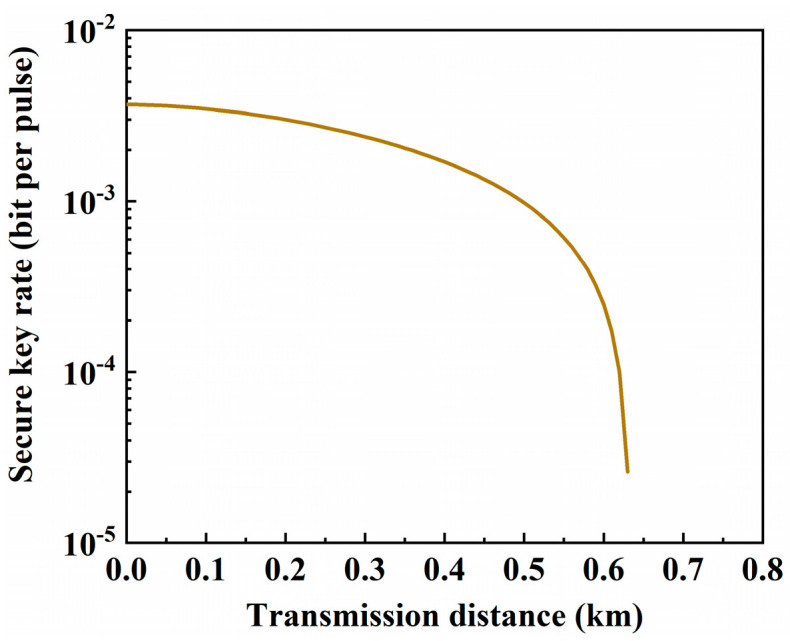
The relationship between secure key rate (SKR) and transmission distance in SMF.

On the basis of the above derivation, the performance of QKD at multiple wavelengths in SMF is demonstrated. In simulation, the quantum channel is at 1550.12 nm, corresponding to ITU standard channel. The classical channels are 1539.77~1542.14 nm with a wavelength interval of 0.8 nm. *η_mn_* between the quantum channel and classical channels can be obtained from Ref. [15]. The commercial SMF used is a step-index fiber, with the structure shown in Figure 1a and parameters listed in Table 1. The power of classical signals is set to 0 dBm, and the power of the quantum signal is −80 dBm. According to Equation (15), the maximum transmission distance of the quantum signal is only 0.64 km due to the interference of SpRS, as shown in Figure 2. In order to increase the transmission distance, the coexistence system is applied in FMF.

As can be seen from Equations (11)–(14), the power of SpRS in FMF is related to the fiber length and the initial power of the signal, *α* and *h_ij_*. Among them, the parameters determined by FMF itself are *α* and *h_ij_*. The *h_ij_* can be expressed as [28]

(18) $$h_{ij}=\frac{2d_{c}{\left|\kappa_{ij}\right|}^{2}}{1+{\left[d_{c}(\beta_{i}-\beta_{j})\right]}^{2}}$$

where *β* is the propagation constant. *κ_ij_* is the mode coupling coefficient, which can be calculated as [29]

(19) $$\kappa_{ij}=\frac{k_{0}^{2}}{\beta_{j}}\iint \left(n^{2}-n_{0}^{2}\right)f_{i}^{*}(x,y)f_{j}(x,y)dxdy$$

where (*n*^2^ − *n*_0_^2^) is the refractive index perturbation [30], and *f*(*x*,*y*) is the mode field distribution.

According to Equations (18) and (19), the *h_ij_* of several commercial FMFs is calculated, and the characteristics applied to coexistence systems are analyzed. The fibers used include two kinds of four-mode fiber (FMF_1_ and FMF_2_), RCF and six-mode fiber (6MF). The structures of the fibers are shown in Figure 1a,b, and the parameters are listed in Table 1. The modes supported by each fiber are shown in Figure 1c. Furthermore, the refractive index difference (Δ*n_eff_*) and *h_ij_* between modes are listed in Table 2 and Table 3, respectively. It is worth noting that, considering the practical application, the quantum channel is placed in LP_01_ mode and set to 1550.12 nm, so only the *h_ij_* between LP_01_ and other modes is calculated in Table 3.

**Figure 3:**
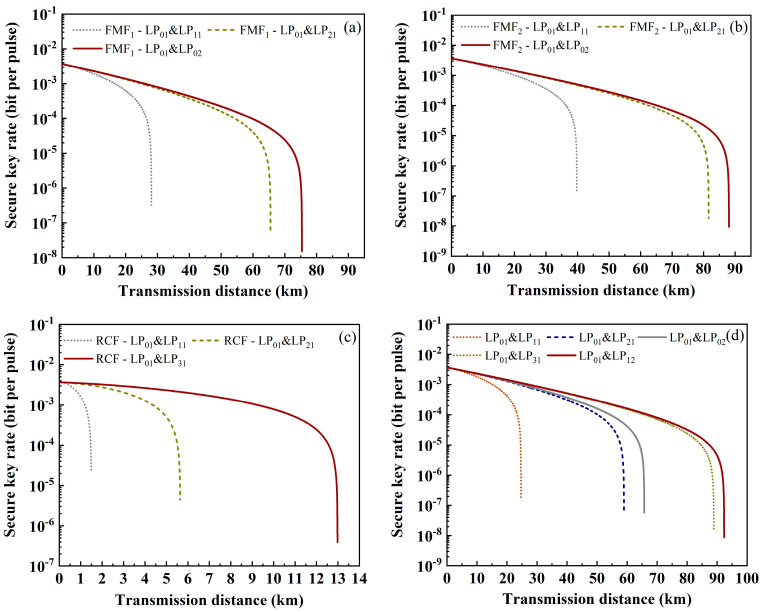
The relationship between SKR and transmission distance for classical signals in single mode. (**a**) FMF_1_, (**b**) FMF_2_, (**c**) RCF, and (**d**) 6MF.

**Figure 4:**
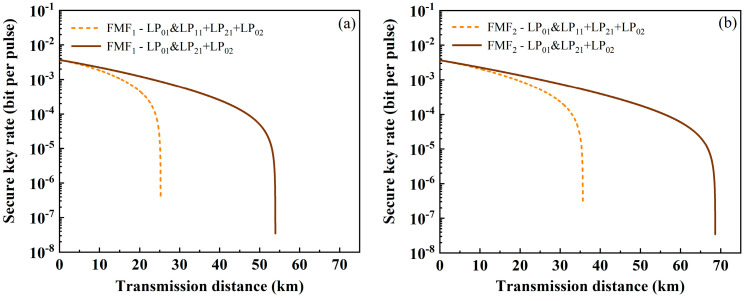

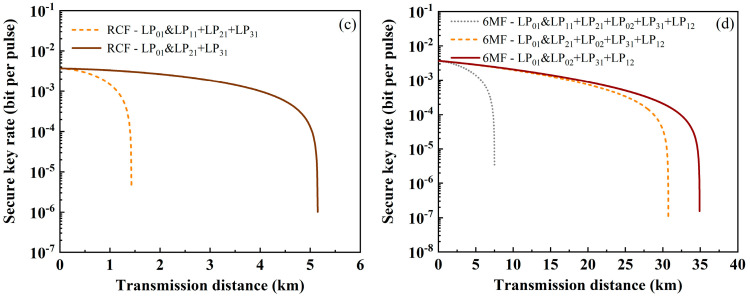
The relationship between SKR and transmission distance for classical signals in multiple modes. (**a**) FMF_1_, (**b**) FMF_2_, (**c**) RCF, and (**d**) 6MF.

Figure 3 and Figure 4 show the relationship between SKR and transmission distance in a coexistence system based on FMF, where the quantum channel is LP_01_ and the classical channels are other modes. WDM technology is used in each classical channel, which can support four wavelengths at the same time. The wavelength range is still 1539.77~1542.14 nm with an interval of 0.8 nm. *η_mn_* can be obtained from Ref. [15]. Referring to the parameters provided on the official website, *α* is set to 0.2 dB/km (LP_01_), 0.2 dB/km (LP_11_), 0.21 dB/km (LP_21_), 0.21 dB/km (LP_02_), 0.215 dB/km (LP_31_) and 0.215 dB/km (LP_12_). Figure 3 shows the variation in SKR in each fiber when the classic channel is only in one mode. Figure 4 is the variation in SKR when the classical signals have multiple modes. Comparing the two sets of figures, it can be seen that the quantum channel will suffer less interference and have a longer transmission distance when there are fewer modes of classical signals. In addition, it is found that when the classical signal is in LP_11_ mode, the transmission distance of the quantum signal will be greatly reduced, which is believed to be caused by the large mode coupling between LP_01_ and LP_11_.

According to previous research, mode coupling in FMFs is related to Δ*n_eff_*. As Δ*n_eff_* increases, Δ*β* also increases, resulting in a decrease in *h_ij_*. Due to the fact that LP_01_ and LP_11_ are adjacent modes, the mode coupling is more pronounced. As reflected in Table 2 and Table 3, *h_ij_* is at a low level when the Δ*n_eff_* is large. Therefore, in order to reduce the power of SpRS in coexistence systems and promote the performance of QKD, it is necessary to reduce *h_ij_*, that is, increase the Δ*n_eff_* to a certain extent. Based on the above discussion, the optimization of FMF will be developed in the following section.

## 4. Performance of QKD in the Ring-Assisted FMF

The most intuitive way to improve Δ*n_eff_* is to increase the effective refractive index difference between core and cladding. However, for the given number of modes and parameters, a large Δ*n_eff_* means a small mode field area, making it difficult to suppress nonlinear effects. The trade-off between mode field area and Δ*n_eff_* in step-index fiber limits the overall performance [31]. In order to solve this contradiction, a ring-assisted FMF was designed [32,33]. The *n_eff_* of modes is controlled by adding a refractive index ring in the fiber core. By carefully selecting parameters of the refractive index ring, the *n_eff_* can be redistributed to optimize the effective refractive index difference between modes.

**Figure 5:**
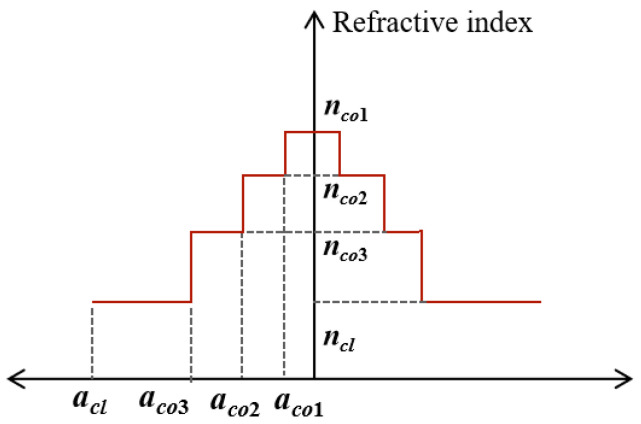
The radius and refractive index distribution of the ring-assisted FMF.

As shown in Figure 3 and Figure 4, the RCF has a large *h_ij_* due to its structure characteristics, resulting in a short transmission distance. Six-mode fiber has difficulties in mode multiplexing and de-multiplexing in practical applications. In four-mode fiber, the transmission distance is longer, but there is also a large mode coupling between LP_01_ and LP_11_. Therefore, we used FMF_2_ as the basic parameter for design, where *a_co_*_3_ = 6 μm, *a_cl_* = 62.5 μm, *n_cl_* = 1.444 and *n_co_*_1_ = 1.459. *a_co_*_1_ and *a_co_*_2_ are the radii of the refractive index rings, while *n_co_*_2_ and *n_co_*_3_ are the refractive indices of the two refractive index rings, as shown in Figure 5.

**Figure 6:**
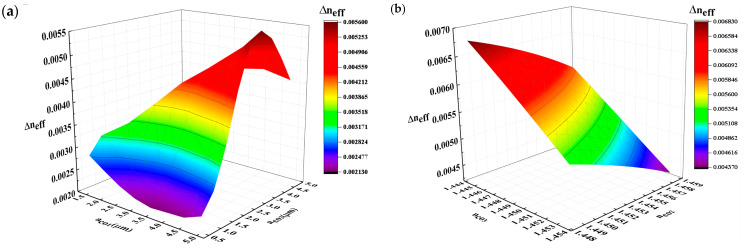
(**a**) The variation in Δ*n_eff_* with *a_co_*_1_ and *a_co_*_2_. (**b**) The variation in Δ*n_eff_* with *n_co_*_2_ and *n_co_*_3_.

Considering the changes involving multiple parameters, the ergodic method was adopted for parameter design. Figure 6 shows the impact of variation in the refractive index ring radius and refractive index on Δ*n_eff_*. The radius ranges are 0.5~4.5 nm and 1.5~5 nm with an interval of 0.5 nm. The refractive index range is 1.445~1.454 with an interval of 0.001. According to the simulation results, the ring-assisted FMF parameters can be determined as *a_co_*_2_ = 5 μm, *a_co_*_1_ = 3.5 μm, *n_co_*_2_ = 1.457, and *n_co_*_3_ = 1.45. Correspondingly, the Δ*n_eff_* and *h_ij_* of the ring-assisted FMF are listed in Table 4. Compared with the parameters in Table 2 and Table 3, the Δ*n_eff_* between LP_01_ and LP_11_ increases, while *h_ij_* decreases significantly.

**Figure 7:**
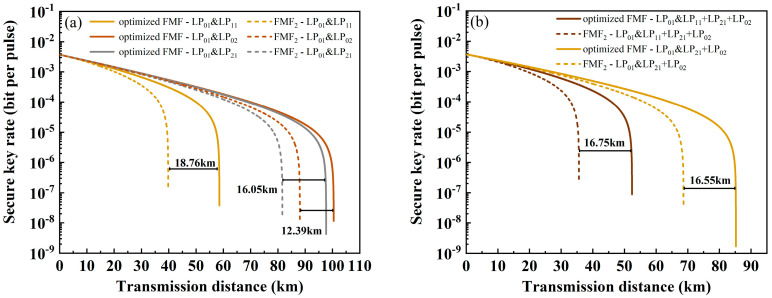
The relationship between SKR and transmission distance when using ring-assisted FMF (solid line) and FMF_2_ (dashed line) with four wavelengths in each mode. Among them, the classical channel is (**a**) one mode and (**b**) multiple modes.

Then, the performance of QKD when using the ring-assisted FMF is calculated. Through WDM technology, each mode of the classical channel still contains four wavelengths. When the classic channel is only one mode, the variation between SKR and transmission distance is as shown in Figure 7a. The solid line represents the ring-assisted FMF, and the dotted line represents FMF_2_. It can be seen that the transmission distance has been greatly improved, with the classical channel showing the greatest improvement at LP_11_, reaching 58.54 km and showing an increase of 47.16%. When the classical channels are LP_21_ and LP_02_, the transmission distance of the quantum signal is increased by 23% and 10.9%, reaching 97.66 km and 100.42 km. Similarly, Figure 7b shows the variation in SKR with transmission distance when the classical channels have multiple modes. When it contains three modes (LP_11_, LP_21_, LP_02_) or two modes (LP_21_, LP_02_), the transmission distance increases by 47% (up to 52.39 km) and 24.1% (up to 85.21 km), respectively.

**Figure 8:**
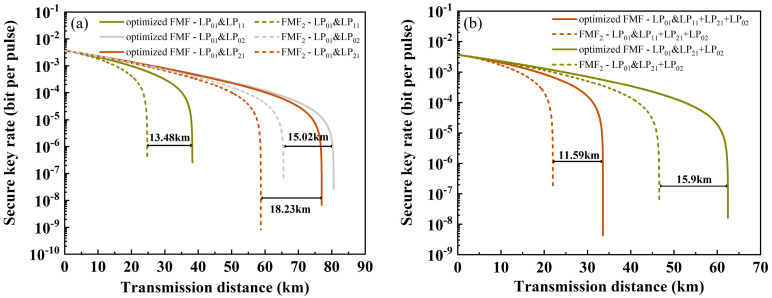
The relationship between SKR and transmission distance when using ring-assisted FMF (solid line) and FMF_2_ (dashed line) with ten wavelengths in each mode. Among them, the classical channel is (**a**) one mode; (**b**) multiple modes.

In Figure 8, the number of wavelengths in each mode of classical channels increases to ten, ranging from 1539.77 to 1546.92 nm with an interval of 0.8 nm. There is a significant variation in transmission distance at different numbers of wavelengths. Figure 8a shows the comparison of ring-assisted FMF and FMF_2_ performance in one mode. When the classic channels are LP_11_, LP_21_ and LP_02_, the transmission distance increases by 54.5%, 37.15% and 17.43%, reaching distances of 38.21 km, 76.97 km and 80.56 km, respectively. Furthermore, when the classical channels include three modes (LP_11_, LP_21_, LP_02_) or two modes (LP_21_, LP_02_), the performance comparisons are as shown in Figure 8b, where the transmission distances increase by 52.68% (up to 33.59 km) and 34.13% (up to 62.48 km).

**Figure 9:**
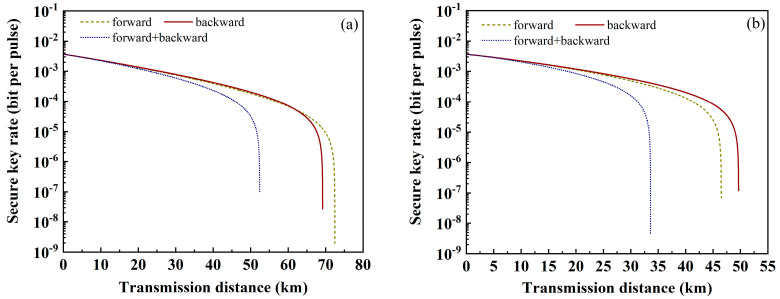
The relationship between SKR and transmission distance when using ring-assisted FMF with (**a**) four wavelengths and (**b**) ten wavelengths in each mode.

The impact of forward and backward SpRS on SKR is also discussed in Figure 9 when using ring-assisted FMF. When there are four wavelengths in each mode, backward SpRS has more impairment of QKD, resulting in a shorter transmission distance. Increasing to ten wavelengths, forward SpRS causes greater damage. The number of wavelengths and channel modes can be selected according to actual applications.

## 5. Conclusions

In summary, this paper derives a simulation model of SpRS in FMF and discusses the impact of SpRS on QKD. The simulation results show that the influence of SpRS depends on the *h_ij_* between modes. On this basis, a ring-assisted FMF is designed to effectively improve the transmission distance of signals in the coexistence system. Furthermore, the impact of forward and backward SpRS on SKR is also discussed. In practical applications, the number of wavelengths and channel modes can be selected according to the requirements.

## Figures and Tables

**Table 1:** The radius and refractive index of each fiber.

	*n_co_*	*n_cl_*	*a_cl_* (μm)	*a_co_* (μm)
SMF	1.452	1.444	62.5	3
FMF_1_	1.452	1.444	62.5	7
FMF_2_	1.459	1.444	62.5	6
RCF	1.459	1.444	62.5	*a_co_*_1_ = 4, *a_co_*_2_ = 7.5
6MF	1.456	1.444	62.5	8

**Table 2:** The Δ*n_eff_* of the FMFs.

Δ*n_eff_* (×10^−3^)						
	LP_01_ and LP_11_	LP_11_ and LP_21_	LP_21_ and LP_02_	LP_31_ and LP_21_	LP_02_ and LP_31_	LP_31_ and LP_12_
**FMF_1_**	2.4	2.9	0.6	-	-	-
**FMF_2_**	3.5	4.5	1.2	-	-	-
**RCF**	0.7	2.1	-	3.1	-	-
**6MF**	2.1	2.8	0.9	-	2.4	1.6

**Table 3:** The *h_ij_* of the FMFs.

	*h_ij_* (km^−1^)				
	LP_01_ and LP_11_	LP_01_ and LP_21_	LP_01_ and LP_02_	LP_01_ and LP_31_	LP_01_ and LP_12_
**FMF_1_**	3.72 × 10^−4^	5.40 × 10^−5^	3.55 × 10^−5^	-	-
**FMF_2_**	1.7962 × 10^−4^	2.7054 × 10^−5^	2.0182 × 10^−5^	-	-
**RCF**	1.41 × 10^−1^	9.14 × 10^−3^	-	1.74 × 10^−3^	-
**6-MF**	4.8449 × 10^−4^	7.1666 × 10^−5^	5.3517 × 10^−5^	1.9382 × 10^−5^	1.6384 × 10^−5^

**Table 4:** The Δ*n_eff_* and *h_ij_* of the ring-assisted FMF.

	Δ*n_eff_* (×10^−3^)			*h_ij_* (km^−1^)		
	LP_01_ and LP_11_	LP_11_ and LP_02_	LP_21_ and LP_02_	LP_01_ and LP_11_	LP_01_ and LP_02_	LP_01_ and LP_21_
**ring-assisted FMF**	5.4	4.2	0.5	6.83 × 10^−5^	9.27 × 10^−6^	1.01 × 10^−5^

## Data Availability

The data presented in this study are available on request from the corresponding author.
